# Efficacy and toxicities of combination maintenance therapy in the treatment of advanced non-small-cell lung cancer: an up-to-date meta-analysis

**DOI:** 10.1042/BSR20182464

**Published:** 2019-06-14

**Authors:** Jianming Hu, Jiawei Hu, Xiaolan Liu, Long Li, Xue Bai

**Affiliations:** 1 Respiratory Department, 1st Hospital of Lanzhou University, Lanzhou, China; 2 Medical Department, Medical School, University of Jinggangshan, Ji’an, China; 3 The People’s Hospital of Tongwei County, Dingxi, Gansu Province, China

**Keywords:** doublet, maintenance therapy, meta-analysis, randomized controlled trials, single agent

## Abstract

**Background:** Single agent maintenance therapy has been approved for the treatment of advanced non-small-cell lung cancer (NSCLC) due to its potential survival benefits, but whether combined maintenance therapy would improve the survival of advanced NSCLC remains undetermined.

**Methods:** Relevant trials were identified by searching electronic databases and conference meetings. Prospective randomized controlled trials (RCTs) assessing combination maintenance therapy in advanced NSCLC patients were included. Outcomes of interest included overall survival (OS), progression-free survival (PFS), and grade 3–4 toxicities.

**Results:** A total of 1950 advanced NSCLC patients received combination maintenance treatment from six trials were included for analysis. The use of doublet maintenance therapy in NSCLC patients significantly improved PFS (HR 0.74, 95%CI: 0.59–0.93, *P =* 0.010), but not for OS (HR 0.95, 95%CI: 0.85–1.07, *P* = 0.40) in comparison with single agent maintenance therapy. Similar results were observed in sub-group analysis according to treatment regimens. In addition, there was no significantly risk difference between doublet and single agent maintenance therapy in terms of grade 3/4 hematologic and non-hematologic toxicities.

**Conclusion:** The findings of the present study show that doublet combination maintenance therapy is superior to single agent maintenance therapy in terms of PFS, without increased grade 3–4 toxicities. Future prospective studies are recommended to clearly assess the long-term clinical benefit of doublet maintenance therapy and its impact on health-related quality of life.

## Introduction

Lung cancer remains one of the most common malignancies in the world and is the leading cause of cancer-related deaths worldwide, accounting for 1.59 million deaths yearly [1]. Non-small-cell lung cancer (NSCLC) accounts for 80–85% of lung cancer cases, which could be further divided into several subgroups, such as adenocarcinoma, squamous cell carcinoma, large cell carcinoma, and others. Generally, NSCLC is often diagnosed at advanced stages when treatment options are limited. Until now, platinum-based doublet chemotherapy remains the standard of care for advanced NSCLC with good performance status, especially in those with tumors that are negative for sensitizing *EGFR* mutations, *ROS-1* and *ALK* [2]. However, most patients will experience disease progression during or after first-line chemotherapy demonstrating the need for new, effective agents or treatment strategy [3].

Maintenance therapy may prolong the time to disease progression and potentially increase overall survival (OS). As a result, maintenance therapy with different drugs is one strategy that has been extensively evaluated in recent years [4–6]. Indeed, several published meta-analyses have confirmed that single agent maintenance therapy in advanced NSCLC prolong the time to disease progression and potentially increase OS in comparison with placebo [7–9]. To date, maintenance therapy with pemetrexed or erlotinib has demonstrated improved OS, resulting in US Food and Drug Administration approval for this indication [6]. Recently, doublet combination maintenance therapy has been investigated in multiple prospective clinical trials, but the results are controversial. As a result, we conduct the present meta-analysis of all available randomized controlled trials (RCTs) to determine the overall efficacy and toxicities of doublet maintenance therapy in advanced NSCLC patients.

## Materials and methods

### Data source

Several databases including PubMed, Web of Science, and Cochrane library were searched for relevant trials. The search key words were maintenance therapy, erlotinib, gefitinib, pemetrexed, gemcitabine, targeted agents, NSCLC, clinical trials, and ovarian cancer. Additionally relevant articles in the reference lists of recent meta-analyses that investigated maintenance therapy in NSCLC patients were also searched. In order to avoid duplication, only the most complete, recent was considered for analysis. All results were input into Endnote X8 reference software (Thomson Reuters, Stamford, CT, U.S.A.) for duplication exclusion and further reference management.

### Study selection

Clinical trials that met the following criteria were included: (1) prospective randomized controlled phase II or III trials involving NSCLC patients; (2) randomized clinical trials comparing doublet versus single agent maintenance therapy; and (3) available survival and toxicities data regarding maintenance therapy in NSCLC patients. If multiple publications of the same trial were retrieved or if there was a case mix between publications, only the most recent publication (and the most informative) was included.

### Data extraction

Two independent investigators conducted the data abstraction, and any discrepancy between the reviewers was resolved by consensus. The following information was extracted for each study: first author’s name, year of publication, trial phase, number of enrolled subjects, treatment arms, maintenance arms, median age, median progression-free survival (PFS), and median OS.

### Outcome measures

A formal meta-analysis was conducted using Comprehensive Meta Analysis software (Version 2.0). The outcome data were pooled and reported as hazard ratio (HR). The primary outcome of interest was OS and secondary outcomes PFS or severe toxicities in NSCLC patients receiving maintenance therapy. Toxicities were defined by the National Cancer Institute’s Common Terminology Criteria for Adverse Events (CTC-AE) during a clinical trial as a result of exposure to an experimental drug, which had been widely used in cancer clinical trials [10]. Between-study heterogeneity was estimated using the χ^2^-based Q statistic [11]. Heterogeneity was considered statistically significant when *P*_heterogeneity_ < 0.1. The presence of publication bias was evaluated by using the Begg and Egger tests [12,13]. A statistical test with a *P*-value less than 0.05 was considered significant. Study quality was assessed by using the Jadad scale based on the reporting of the studies’ methods and results [14].

## Results

### Search results

**Figure 1:**
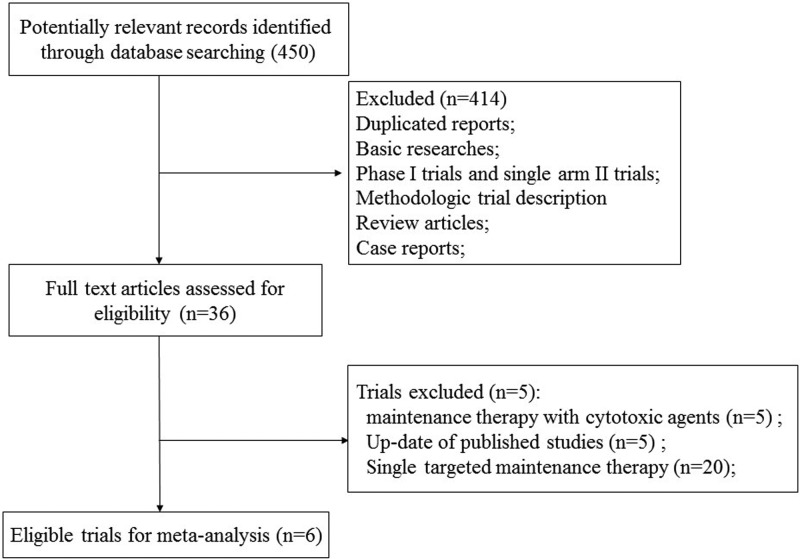
Studies eligible for inclusion in the meta-analysis

**Figure 2:**
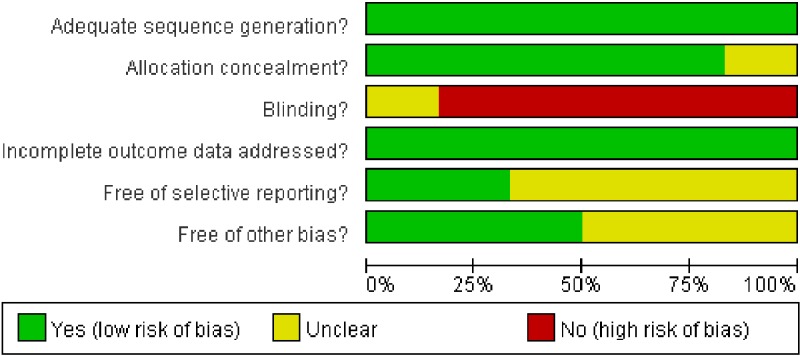
Random-effect model of hazard ratio (95%CI) of PFS in NSCLC treated doublet versus single agent maintenance therapy

We performed the systematic review and meta-analysis in accordance with the Preferred Reporting Items for Systematic Reviews and Meta-Analysis (PRISMA) statement [15]. Our initial search yielded 450 potentially relevant reports. After excluding review articles, phase I studies, case reports, meta-analyses, and observation studies, a total of seven prospective randomized controlled clinical trials were included. After reviewing of included trials, two included trials were undated analysis of previously published trials [16,17], and the most recent publication (and the most informative) was included [17]. Finally, a total of 1950 advanced NSCLC patients from six trials were included for analysis (Figure 1) [17–22]. The search strategy was listed in Supplementary material. Table 1 listed the baseline characteristics of patients and studies. The quality of each included study was roughly assessed according to Jadad scale, and two of the six RCTs were double-blind placebo-controlled trials, thus had Jadad score of 5. Other three trials were an open-label controlled trial, thus had Jadad score of 3. The risk of bias assessment of the included RCTs was shown in Figure 2. And the risk of bias assessment of included trials was low.

**Table 1:** Baseline characteristics of six included trials

Authors/years	Population	Induction therapy	Treatment group	Maintenance regimen	No. of patients	Median age	Median PFS	Median OS	Jadad score
**Barlesi F. et al./2013**	CT-naïve, Stage IIIB–IV, non-squamous, ECOG PS 0–2	Pemetrexed +cisplatin +bevacizumab	Experimental arm (doublet)	Bevacizumab +pemetrexed	128	NR	7.4 (0.48, 0.35–0.66	19.8 (0.88, 0.63–1.21)	3
			Control arm (single agent)	Bevacizumab	125	NR	3.7	15.9	
**Johnson B.E. et al./2013**	CT-naïve, Stage IIIB–IV, or recurrent, ECOG PS 0–1	Chemotherapy +bevacizumab	Experimental arm (doublet)	Bevacizumab erlotinib	370	64	4.8 (0.71, 0.58–0.86	14.4 (0.92, 0.70–1.21)	5
			Control arm (single agent)	Bevacizumab placebo	373	64	3.7	13.3	
**Patel J.D. et al./2013**	CT-naïve, non-squamous, Stage IIIB–IV, or recurrent, ECOG PS 0–1	Chemotherapy +bevacizumab	Experimental arm (doublet)	Bevacizumab +pemetrexed	292	63.8	6 (0.73, 0.71–0.96	12.6 (1, 0.86–1.16)	3
			Control arm (single agent)	Bevacizumab	298	64.3	5.6	13.4	
**Karayama M. et al./2016**	CT-naïve, non-squamous, Stage IIIB–IV, or recurrent, ECOG PS 0–1	Pemetrexed +carboplatin +bevacizumab	Experimental arm (doublet)	Bevacizumab +pemetrexed	45	66	11.5 (0.73, 0.44–1.19	24.4, 0.87, 95% CI: 0.49e1.54	3
			Control arm (single agent)	Pemetrexed	35	65	7.3	21.3	
**Ciuleanu T.E. et al./2017**	CT-naïve, Stage IV, or recurrent, ECOG PS 0–1	Platinum-based chemotherapy	Experimental arm (doublet)	Linsitinib +erlotinib	102	62	125, 1.09 (0.788–1.507)	381, 1.20 (0.777, 1.853)	5
			Control arm (single agent)	Placebo +erlotinib	103	60	129	421	
**Niho S. et al./2017**	CT-naïve, Stage IIIB–IV, or recurrent, ECOG PS 0–1	Platinum-based chemotherapy	Experimental arm (doublet)	S-1+bevacizumab	39	61	4.6 (0.64, 0.45–0.91	19.9 (0.65, 0.41–1.02)	3
			Control arm (single agent)	Bevacizumab	40	65	2.6	11.0	

Abbreviations: CT, chemotherapy; ECOG, Eastern Cooperative Oncology Group; NR, not reported; OS, overall survival; PFS, progression-free survival; PS, performance status.

### Progression free survival

**Figure 3:**
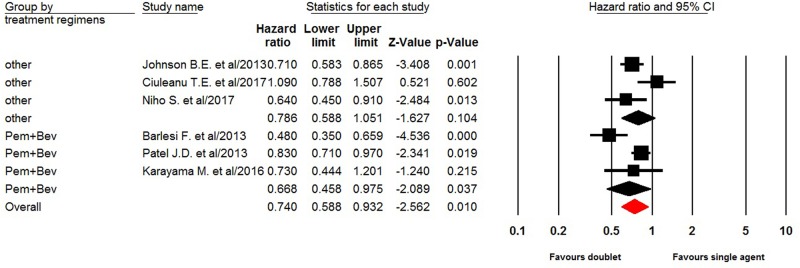
Fixed-effect model of hazard ratio (95%CI) of OS in NSCLC treated doublet versus single agent maintenance therapy

All of six trials reported PFS data of doublet versus single agent maintenance therapy in NSCLC patients. The pooled hazard ratio for PFS demonstrated that the doublet maintenance therapy in NSCLC patients significantly improved PFS giving HR 0.74 (95%CI: 0.59–0.93, *P =* 0.010, Figure 3), in comparison with single agent maintenance therapy. There was significant heterogeneity between trials (*I*^2^ = 67.6%, *P* = 0.009), and the pooled HR for PFS was performed by using random-effect model. Sub-group analysis according to maintenance regimen showed that pemetrexed plus bevacizumab maintenance therapy (HR 0.67, 95%CI: 0.46–0.98, *P* = 0.0037) in NSCLC patients significantly improved PFS in comparison with single agent maintenance therapy, but not for other doublet maintenance therapy (HR 0.79, 95%CI: 0.59–1.05, *P* = 0.104).

### Overall survival

**Figure 4:**
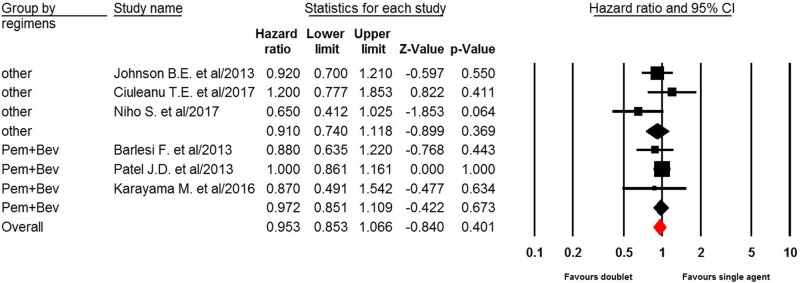
Fixed-effect Model of hazard ratio (95%CI) of OS in NSCLC treated doublet versus single agent maintenance therapy

Six trials reported OS data of doublet versus single agent maintenance therapy in NSCLC patients. The pooled hazard ratio for OS indicated that doublet maintenance therapy in NSCLC patients did not significantly improved OS giving HR 0.95 (95%CI: 0.85–1.07, *P =* 0.41, Figure 4), in comparison with single agent maintenance therapy. There was no significant heterogeneity between trials (*I*^2^ = 0%, *P* = 0.47), and the pooled HR for OS was performed by using fixed-effect model. We then performed sub-group analysis according to maintenance regimens and found that both pemetrexed plus bevacizumab (HR 0.97, 95%CI: 0.85–1.11, *P* = 0.67) and other doublet maintenance therapy agents (HR 0.91, 95%CI: 0.74–1.12, *P* = 0.37) did not significantly improved OS in comparison with single agent maintenance therapy.

### Toxicities

Pooled analysis of reported grades 3 and 4 adverse events (AEs) of interest was also performed. There was no significantly risk difference between doublets and single agent maintenance therapy in terms of grade 3/4 hematologic (anemia, neutropenia, and thrombocytopenia) and non-hematologic toxicities (diarrhea, nausea, and fatigue) (Table 2).

**Table 2:** Outcome of grade 3 or 4 toxicity comparing doublet versus single agent maintenance therapy

Toxicity	Trials	doublet therapy	Single agent	Heterogeneity		RR(95%CI)	*P* value
				*P* value	*I*^2^		
Grade 3–4 Anemia	3	8/212	2/200	0.26	26.0	2.24(0.47-10.6)	0.31
Grade 3–4 neutropenia	3	14/212	3/200	0.11	54.5	3.44(0.45-26.2)	0.23
Grade 3-4 thrombocytopenia	3	1/212	0/200	0.98	0	2.35(0.10-55.9)	0.60
Grade 3–4 Diarrhea	4	43/511	14/516	0.025	73.0	2.23(0.52-9.56)	0.28
Grade 3-4 nausea	4	23/314	12/303	0.74	0	1.71(0.89-3.31)	0.11
Grade 3–4 Fatigue	4	7/314	7/303	0.72	0	0.95 (0.33-2.72)	0.93

### Publication bias

Begg’s funnel plot and Egger’s test were performed to assess the publication bias of the literature. The shapes of the funnel plots did not reveal any obvious asymmetry (*P* = 0.85 for PFS and *P* = 0.45 for OS). Egger’s test was used to provide statistical evidence of funnel plot symmetry. The results suggest no evidence of publication bias for PFS and OS (*P* = 0.60, *P* = 0.38, respectively).

## Discussion

First-line platinum-based chemotherapy of four or six cycles has reached a plateau of effectiveness for the treatment of advanced NSCLC. Unfortunately, the prognosis of these patients is poor, with a 5-year survival less than 5% [23]. Maintenance therapy has emerged as a novel treatment strategy for advanced NSCLC patients. Indeed, multiple studies have demonstrated that single agent maintenance therapy in advanced NSCLC significantly improved PFS and OS in comparison with single agent maintenance therapy. The PARAMOUNT trial conducted by Paz-Ares et al. found that Continuation maintenance with pemetrexed significantly reduced the risk of disease progression over the placebo group (HR 0.62, 95% CI 0.49–0.79; *P*<0.0001). And the authors recommended that pemetrexed maintenance is an effective and well tolerated treatment option for patients with advanced non-squamous NSCLC with good performance status who have not progressed after induction therapy with pemetrexed plus cisplatin [28]. Another two phase III trials evaluating EGFR-tyrosine kinase inhibitors as maintenance therapy for advanced NSCLC patients, and found that erlotinib maintenance therapy significantly improved progress-free survival in comparison with placebo [24–26]. Due to the survival benefits from maintenance therapy, the US FDA approves the use of erlotinib or pemetrexed as maintenance therapy for the treatment of advanced NSCLC patients [24–30]. More recently, with the introduction and dissemination of checkpoint inhibitors, significant improvement had been archived for the treatment of advanced NSCLC patients. The role of immunotherapy maintenance therapy in NSCLC has been also investigated in NSCLC. The PACIFIC trial [31] was a Phase III double-blind randomized placebo-controlled trial. Patients who did not progress following definitive platinum-based chemotherapy (≥2 cycles) concurrent with radiotherapy were enrolled. Patients were randomized in a 2:1 fashion to 10 mg/kg of durvalumab every 2 weeks versus a similarly administered placebo. The median PFS was 5.6 months in the placebo arm and 16.8 months in the durvalumab arm. In addition to the impressive PFS data, the ORR was significantly higher in the durvalumab arm than in the placebo arm (28.4% versus 16%, respectively; *P* = 0.001). However, to our best knowledge, whether doublet combination therapy would improve efficacy in comparison with single agent maintenance therapy remains undetermined.

In the preset meta-analysis, a total of 1950 advanced NSCLC patients from six trials are included. Our results show that doublet maintenance therapy in NSCLC patients significantly improves PFS (HR 0.73, 95%CI: 0.60–0.89, *P =* 0.002), but not for OS (HR 0.95, 95%CI: 0.85–1.07, *P* = 0.40) in comparison with single agent maintenance therapy. We then perform sub-group analysis according to maintenance regimens, and find that pemetrexed plus bevacizumab maintenance therapy significantly improve PFS, but not for OS. In addition, the toxicities of doublet combination maintenance therapy are minimal and well tolerated. There is no significantly risk difference between doublet and single agent maintenance therapy in terms of grade 3/4 hematologic and non-hematologic toxicities.

Give only modest improvement in PFS or OS obtained from maintenance therapy, quality of life (QOL) is another issue needed to be concerned for patients and physicians. However, none of the included trials report the result of QOL between doublets versus single agent maintenance therapy in NSCLC patients. Several single agent maintenance trials incorporate QOL analysis into their design and find that QOL is not significantly worse with maintenance therapy and may delay the time to pain or other disease-related symptoms [29,32]. As a result, future studies are recommended to investigate the impact of doublet combination maintenance therapy on health-related QOL.

There are several limitations exist in this analysis. First, this meta-analysis only includes published trials, and a meta-analysis of individual level data might define more clearly treatment benefits in specific subgroups. Secondly, different doublet combination maintenance regimen are included for analysis in the present study, which might increase the heterogeneity among included trials. In addition, we could not answer which regimen is the best choice. Thirdly, the optimal timing and duration of maintenance therapies using different targeted agents are still needed to be defined in further studies. Finally, publication bias is an important issue for meta-analysis because trials with positive results are more likely to be published. Our paper does not detect publication bias for PFS and OS.

## Conclusions

Our study suggests that doublet maintenance therapy in advanced NSCLC patients demonstrates a PFS benefits, but not for OS benefits in comparison with single agent maintenance therapy. In addition, doublet maintenance therapy does not significantly increase the risk of severe toxicities when compared with single agent maintenance therapy. Future trials are suggested to assess the long-term clinical benefit of doublet maintenance treatment in NSCLC patients and its impact on health-related QOL.

## Abbreviations

NSCLC: non-small-cell lung cancer
OS: overall survival
PFS: progression-free survival
QOL: quality of life
RCT: randomized controlled trial
